# Feedback on clinical team performance: how does it work, in what contexts, for whom, and for what changes? A critical realist qualitative multiple case study

**DOI:** 10.1186/s12913-023-09402-x

**Published:** 2023-04-27

**Authors:** Joachim Rapin, Sylvie Gendron, Cédric Mabire, Carl-Ardy Dubois

**Affiliations:** 1 Faculty of Nursing, Université de Montréal, 2375 Chemin de la Côte-Sainte-Catherine, QC H3T 1A8 Montréal, Canada; 2 Lausanne University Hospital, Rue du Bugnon 21, 1011 Lausanne, CH Switzerland; 3 Institute of Higher Education and Research in Healthcare – IUFRS, University of Lausanne, Biopôle 2 – Route de la Corniche 10, 1010 Lausanne, CH Switzerland; 4 École de Santé Publique de l’Université de Montréal, 7101 Avenue du Parc, QC H3N 1X9 Montréal, Canada

**Keywords:** Nurses, Clinical audit, Feedback, Quality improvement, Performance measurement, Qualitative research, Theory, Learning health system, Comparative study

## Abstract

**Background:**

Feedback on clinical performance aims to provide teams in health care settings with structured results about their performance in order to improve these results. Two systematic reviews that included 147 randomized studies showed unresolved variability in professional compliance with desired clinical practices. Conventional recommendations for improving feedback on clinical team performance generally appear decontextualized and, in this regard, idealized. Feedback involves a complex and varied arrangement of human and non-human entities and interrelationships. To explore this complexity and improve feedback, we sought to explain how feedback on clinical team performance works, for whom, in what contexts, and for what changes. Our goal in this research was to present a realistic and contextualized explanation of feedback and its outcomes for clinical teams in health care settings.

**Methods:**

This critical realist qualitative multiple case study included three heterogeneous cases and 98 professionals from a university-affiliated tertiary care hospital. Five data collection methods were used: participant observation, document retrieval, focus groups, semi-structured interviews, and questionnaires. Intra- and inter-case analysis performed during data collection involved thematic analysis, analytical questioning, and systemic modeling. These approaches were supported by critical reflexive dialogue among the research team, collaborators, and an expert panel.

**Results:**

Despite the use of a single implementation model throughout the institution, results differed on contextual decision-making structures, responses to controversy, feedback loop practices, and use of varied technical or hybrid intermediaries. Structures and actions maintain or transform interrelationships and generate changes that are in line with expectations or the emergence of original solutions. Changes are related to the implementation of institutional and local projects or indicator results. However, they do not necessarily reflect a change in clinical practice or patient outcomes.

**Conclusions:**

This critical realist qualitative multiple case study offers an in-depth explanation of feedback on clinical team performance as a complex and open-ended sociotechnical system in constant transformation. In doing so, it identifies reflexive questions that are levers for the improvement of team feedback.

**Supplementary Information:**

The online version contains supplementary material available at 10.1186/s12913-023-09402-x.

## Background

Feedback on clinical team performance is a strategy that aims to provide clinical teams in health care settings with structured results (e.g., process or outcome indicators, or even structure) regarding their performance [1]. Conventional knowledge suggests that this strategy encourages health professionals to change their behavior in order to adopt desired clinical practices. A systematic review of 140 randomized studies suggested that feedback improved compliance with desired clinical practices (risk-adjusted weighted median: 4.3%), although there was marked variability across settings (interquartile range: 0.5-16%) [2]. These results are similar to those of a systematic review that included seven randomized studies [3]. This variability could be explained by various contextual conditions, inconsistent feedback processes [2], or the way in which two or more people or things are connected and affect one another (i.e., sociotechnical components) [3].

To improve feedback on clinical team performance, traditional recommendations take the form of lists, sometimes containing more than 300 items [4–6]. More recently, a consensus based on the opinion of 68 experts identified seven priority explanatory hypotheses:

“(1) The feedback is provided by a trusted source; (2) recipients are involved in the design/development of the feedback intervention; (3) recommendations related to the feedback are based on good quality evidence; (4) behaviour is under the control of the recipient; (5) it addresses barriers and facilitators to behaviour change; (6) it suggests clear action plans; (7) target/goal/optimal rates are clear and explicit.” ([7], p. 5).

Most of these explanatory hypotheses seem to be decontextualized or even idealized. In the health care system, power and knowledge are shared between different actors with different values, priorities, and interests [8]. Moreover, their values, priorities, and interests may change over time. For example, care or treatment provided to a patient can be negotiated or adapted, depending on patient preferences, health conditions, professional experiences, or organizational constraints, and can deviate from recommendations. In other words, the behavior of an individual or a team can adapt or adjust to interactions and contexts. Current studies hardly mention contextual mechanisms that explain the variability in observed impact as a consequence of feedback interventions [5, 6].

Feedback on clinical team performance involves social and technical interactions in a specific evolving context. For example, public release of results may cause professionals to take action to improve patient care for fear of damage to their reputation [9]. They may focus on indicators to the detriment of the clinic (tunnel vision). Analysis of indicators can generate controversies on possible causes or solutions. In feedback, actors have a high degree of autonomy in carrying out the processes [10, 11]. At the same time, these actors are constrained by their sociotechnical context and interactions. Feedback on clinical team performance is based on an unpredictable and complex interdependent sociotechnical system.

As described previously [12], the current state of research on feedback limits the ability to understand (1) the occurrence and evolution of social interactions during feedback on clinical team performance, within their context; (2) the evolution of sociotechnical interactions, within particular contexts; and (3) the transformations generated by such a complex system of sociotechnical interactions. We postulated that a critical realist qualitative multiple case study would provide answers to these three limits.

### Prior theoretical and empirical work

As a first step in addressing these limitations and providing an initial critical realist theory, we conducted a rapid realist review that resulted in 12 contextualized configurations to explain what feedback is, how it works, and in what contexts it works with clinical teams [12]. These configurations are grouped into three interrelated chronological hypotheses that involve (a) preparatory work for feedback on clinical team performance, (b) feedback processes, and (c) subsequent to feedback, transformations in interrelationships between entities involved in feedback and performance improvement.

The first hypothesis presents a favorable context in which clinical teams participate in the preparatory work of feedback. Participation contributes to their involvement in feedback processes through prior alignment of different expectations, identities, roles, and practices. On the one hand, this participation strengthens connections between different actors. On the other hand, it contributes to their enrolment because it implies adherence, which is a manifestation of their consent to improve care.

The second hypothesis, which refers to feedback on clinical team performance processes per se, describes a supportive environment where trusted and respected professionals introduce intermediaries. For example, a patient’s history or human values (e.g., respect, caring, justice) can support recognition of an issue. As another example, some professionals can produce complementary data to interest colleagues in possible alternative solutions. In this way, they connect and move actors toward the collective recognition of a problem, further negotiations, the identification of possible solutions, or the sustainable mobilization of newfound solutions.

The third hypothesis illustrates how the involvement of clinical teams in feedback processes generates change in feedback practices and, more globally, in the performance improvement system. This engagement strengthens interrelationships between the different actors or entities, for example, by modifying indicators to better meet the expectations of professional teams.

However, our rapid realist review has its limitations. The documents included were not primarily intended to describe the social dimensions of feedback processes, the socio-technical or contextual interrelationships between different actors and entities, or feedback controversies. Thus, a confrontation with the actual practice of feedback was necessary. Moreover, these three hypotheses identify favorable contexts in which teams collectively mobilize for performance improvement. Are these contexts sustainable, or even idealized?

The purpose of this research is to present a real and contextualized theory of feedback on clinical team performance and its outcomes in a health care setting. We ask the following question: how does feedback on clinical team performance work, for whom, in what contexts, and for what changes? Resultant explanations could provide guidance for improving feedback on clinical team performance.

## Methods

### Study design: a theoretically driven qualitative multiple case study

In this qualitative research, we relied on multiple case study methodology [13, 14] that combines theoretical parameters derived from Critical Realism (CR) [15] and Actor-Network Theory (ANT) [16, 17] to explain the real world, which is complex and composed of varied entities (human and non-human) and interrelationships. In CR terms, rigorous scientific research attempts to provide convincing explanations of real-world entities, their interrelationships, and the outcomes they generate. These explanations provide a refined theory that is transitive, as the real world is constantly changing. The RAMESES II reporting standards for realist evaluations is provided in Additional file 1 [18].

Specifically, we relied on the CR configuration referred to as *Context and Mechanism(s) = > Outcome* (C & M(s) = > O) [19] to explore and delineate how context interacts with one or more mechanisms to produce outcomes. We then defined and refined this C & M(s) = > O configuration to conceptualize feedback on clinical team performance and its outcomes with reference to concepts that are fundamental to ANT. In particular, we referred to ANT conceptualization of different entities that are present and interrelated in the real world, as well as their specific functions: intermediaries (e.g., documents, values, tools, resources, or skills that give meaning to the network), actors (human or non-human who use or produce intermediaries), mediators (actors that move or obstruct other actors), and the networks that connect these different entities. Otherwise, the four translation operations that generate outcomes, as defined by ANT, shaped our approach to the study object: (1) problematization (some actors are or become connected as they interact around emerging problems or issues that emerge with feedback), (2) interessement (some actors change their identities, develop strategies, engage and connect, or displace other actors to solve problems or issues), (3) enrollment (some actors define and interconnect their roles to match their interests), and (4) mobilization (a critical mass of actors becomes capable of coordinating their efforts to act together). As well, ANT conceptualizes outcomes as actions distributed among human and non-human actors [20].

**Fig. 1:**
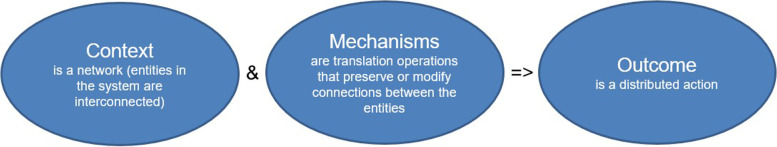
Model of the ANT-CR combination. Reproduced with permission from Springer Nature [12]

Figure 1 represents the CR and ANT combination driving this study. Context is conceptualized as a network of interconnected entities in an open system; mechanisms are translations that maintain or change connections between entities; and outcomes are distributed actions. Overall, this model suggests that if some entities are interconnected in a feedback on clinical team performance system (context), then some of them could perform distributed actions (outcome) because translation operations (mechanisms) maintain or change the connections between the entities.

Finally, this theoretically driven qualitative multiple case study, conducted over a period of 36 months (01.01.2020 to 22.12.2022), was also designed in keeping with lessons derived from CR evaluation research, most notably that “spaces of mechanisms” should first be identified to initiate investigations [21]. With reference to ANT applied to our study, and given our experience of feedback on clinical team performance, the concepts of controversy and mediation were identified as spaces of mechanisms to explore. “Controversies [tie] together and enmesh the techno-scientific and political contents that make up the issues facing actors” ([22], p. 176). Mediation refers to the actions of mediators that tend to move in a network or elsewhere other actors.

### Study site

The Centre Hospitalier Universitaire Vaudois (CHUV), located in Lausanne, Switzerland, has 1,400 beds across several sites, and offers acute care, rehabilitation, outpatient, and long-stay facilities serviced by more than 12,000 professionals. Over 4,900 nurses, care and community health assistants (i.e., patient attendants), physiotherapists, and occupational therapists are attached to the Care Directorate.

In 2012, this directorate mandated a team to develop a nursing performance improvement system, now implemented in over 50 clinical units. This system produces periodic reports pertaining to 12 structure, process, and outcome indicators via an interactive dashboard accessible to health care team managers (e.g. skill mix in clinical teams, absenteeism, urinary infections from catheter use, pressure ulcer prevention and rates, pain prevention and management). Local managers are expected to organize bimonthly meetings with a local performance group (LPG) specific to each clinical unit to analyze, plan, and implement actions designed to improve clinical practice. LPGs are composed of five to seven professionals who participate voluntarily and sometimes on a long-term basis. They include, for example, a nurse supervisor (NS), a head nurse (HN), clinical nurse specialists (CNS), nurses, certified health care assistants, and, rarely, physicians or other professionals.

### Sample

In this study, a case is a clinical unit’s LPG and its network of partners and resources outside the unit that we refer to as a *feedback on clinical team performance system.* Our rapid realist review suggested two criteria for the selection of cases: (a) diversity of contexts, that is, diverse networks of interconnected entities engaged (or not) in feedback processes; and (b) different feedback processes. Two cases are described here as having a narrow context (LPG and unit) and routine feedback processes, whereas the third case comprises a broad context and diverse or innovative processes.

Further heterogeneity required for this in-depth qualitative multiple case study lies in the variety of entities that constitute each case, such as the composition of clinical teams, managers of varied experience, documents, values, and communication tools. For each case, a chain sampling strategy was conducted [23] to ensure the inclusion of networks of entities (including mediators, actors, intermediaries) that were directly connected to the feedback processes examined. Health care professionals who had the ability to inform a particular aspect of the research [13, 23] and managers who were key players in feedback processes were also included. For each case, this comprehensive sampling strategy [23] gave us access to different hierarchical levels of management: HN (first hierarchical level), department NS (second hierarchical level), and department director (third hierarchical level). Finally, the CHUV director of care service (fourth hierarchical level) was also included. JR, SG, CM, and CAD and participants were consulted to assess the appropriateness of this chain sampling method during data collection and analysis. Patients were excluded from this research, as they were not directly connected to the feedback.

### Data collection

Five methods of data collection were used to obtain dense and varied material for an in-depth understanding of each case: participant observation [24–26], document retrieval, focus groups [27–29], semi-structured interviews [27, 30], and questionnaires for focus group participants.

Observations were conducted for 15 days for one case and 6 months for two cases. For the first case, we stopped data collection (06.03.2020) because of the COVID-19 pandemic, as participants were no longer available for this study. All observations were documented with a guide inspired by Decuypere [31] (Additional file 2). Documentary intermediaries retrieved from three cases included, for example, performance reports and graphs, minutes from LPG meetings, action plans, support guides, or electronic reports. All documents were printed prior to analyses. These intermediaries are context entities, that is, they carry information across teams and between actors.

Focus groups and semi-structured interviews were conducted during the first and sixth month of data collection in order to monitor the evolution of participants’ experience and to have as much temporal variation as possible. The interview guide – identical for both methods – was based on our theoretical parameters (Additional file 3). Given that differences in hierarchical status can limit the expression of ideas by some participants [29], individual interviews were conducted with managers, and focus groups were conducted with the other participants. JR conducted the individual semi-directed audio-recorded interviews and was supported by a research assistant to conduct the focus groups, which were also audio-recorded. Inspired by the realist method, the interview guide was designed to identify the roles and interests of key players [13]. Specific informations of the focus group participants was collected a posteriori with a questionnaire (Additional file 4).

Throughout data collection, JR completed a logbook to gather different types of notes [25]. Descriptive notes, completed during and after observation sessions, focus groups, and semi-directed interviews, included, for example, environment, events, interactions, actors, intermediaries, and potential controversies. Methodological notes kept a detailed record of planning, reminders of the stages of the method, or any modifications to the research process. Reflexive and analytical notes are described below.

### Data management, analysis, and synthesis

A research assistant transcribed all audio recordings of interview material. Reliability was ensured by JR, who read the transcripts while listening to the recordings. Participant observation material was transcribed electronically by JR and was uploaded with the interview transcriptions into NVivo software 12.2.0 for data analyses.

**Fig. 2:**
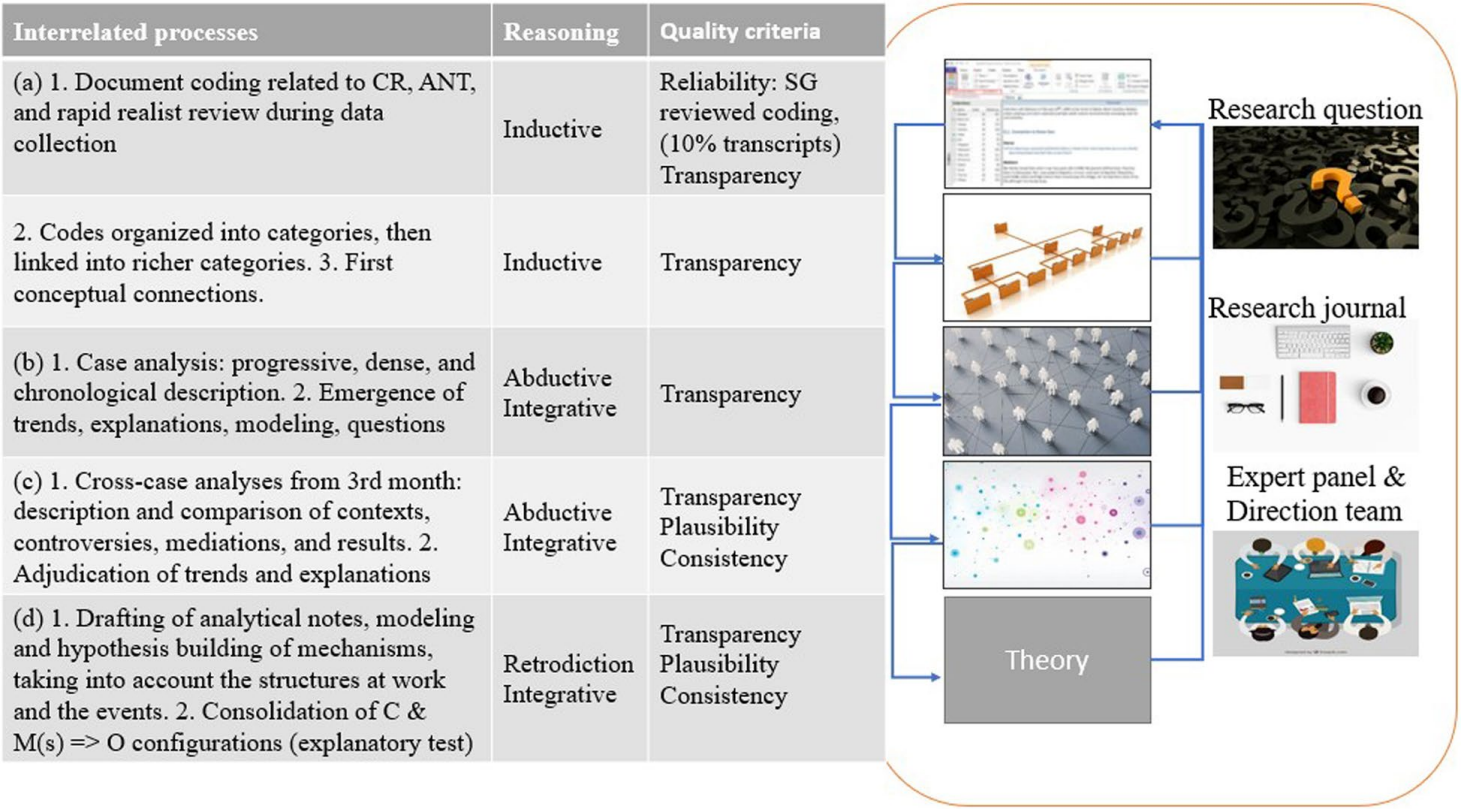
Data analysis and synthesis processes

From data collection to the writing of the final report, an iterative process that comprised concurrent cycles of analysis and synthesis involved the following: (a) coding and conceptual interpretation based on theoretical parameters (Additional file 5) [32, 33], (b) case analysis and initial modeling [34–36], (c) cross-case analysis that brought together the results of the previous two processes [13], and (d) synthesis in the form of a theory [15, 19, 37]. Each process involved a return to transcribed data to support or test interpretations. The analysis focused on cases 2 and 3 because the data were richer and denser. Case 1 data was used to further refine our interpretations. Figure 2 presents each process, specific types of reasoning, and quality criteria.

As analyses proceeded, JR used his logbook to produce analytical and reflexive notes (reflexivity in the sense of an internal and social process). Analytical notes allowed JR to document intuitions, reflections, configurations, or theoretical modeling. Reflexive notes describe JR’s standpoint and its evolution [13]; as well as his integration into the environment, successes, difficulties, emotions, or reactions [25]. Syntheses of these notes were shared with the research team to support JR’s reflexive process.

SG, CM, CAD, and a panel of experts contributed to the data analysis. This panel was composed of four clinical experts from the CHUV and four external experts, three of whom are members of the *Conseil Consultatif sur la qualité des soins et la sécurité des patients du Secrétariat international des infirmières et infirmiers de l’espace francophone* (SIDIIEF). Two meetings were held with the panel and more than 20 with SG, CM, or CAD. Each time, JR presented his reasoning and results; and panel experts and research team members could, for example, propose alternative explanations or question the logic of the data analyses and the synthesis of results [38, 39].

### Quality criteria

Four realist method quality criteria are referred to in this study: plausibility, coherence, trustworthiness, and transparency [39]. Plausibility refers to the best plausible resultant theory. To judge plausibility, JR tested some ideas with participants, submitted his interpretations to the expert panel, and shared data-based resultant theories with SG, CM, and CAD. Adjudication was further verified with reference to coherence, defined by Wong [39] as follows:

“consilience (or explanatory breadth) – the ability of the theory to explain as much as possible of the data; simplicity – the theory is simple and does not have to have special (or ‘ad hoc’) assumptions made to it to explain data; analogy – the theory fits in with what we currently know and/or substantive theory.” (p. 179).

Regarding trustworthiness, a realist theory is based on multiple events and experiments that are part of a complex reality conveyed through various data collection and analysis methods combined, as well as methodical synthesis of results [39]. Trustworthiness requires the demonstration that a scientific method has been rigorously applied and, specifically, that relevant data collection has been conducted multiple times in order to produce the theory.

Transparency involves extensive documentation of cases, research procedures, and the researcher’s standpoint to ensure reliability, quality of reasoning, and procedural accountability. Reliability and transparency were judged through the ongoing documentation of the logbook that was discussed with the research team.

## Results

### Sample characteristics

Ninety-eight professionals participated in this study. Table 1 presents participant distribution by professional role and by case.

**Table 1:** Distribution of study participants by professional role and by case

Professional function	Case 1	Case 2	Case 3	Actors across cases	Total study participants
Nurse manager	5	10	6	3	24
Clinical nurse	5	20	6	NA^a^	31
Certified health care assistant	4	16	5	NA^a^	25
Physician manager	0	3	2	NA^a^	5
Physician	0	0	1	NA^a^	1
Other professionals	0	6	0	NA^a^	6
Student and apprentice	0	3	3	NA^a^	6
Total	14	58	23	3	98

^a^*NA *Not applicable

The shortened data collection period explains the small sample in case 1. In case 2, the clinical team, feedback network, and diversity of professions were larger than in case 3. For case 2 and 3, each profession involved in feedback and care is represented in our sample. Some actors are present in all 3 cases. These actors across cases are members of the Care Directorate and are engaged in the three cases. In Switzerland, nurses and certified health care assistants are distinct professions.

### Data collection

Overall, 120.5 h of participant observation were conducted within 41 periods, and 26 individual interviews spanned over 37.5 h. As well, four focus groups generated 15 socio-demographic questionnaires. Throughout data collection, 82 documents were retrieved.

Table 2 presents the distribution of data sources per case.

**Table 2:** Distribution of data sources by case

Data type	1st case	2nd case	3rd case	Actors and documents across cases	Total
Observation	18.75 h	70 h	31.75 h	NA^a^	120.5 h
Individual interviews	2	5	11	8	26
Focus groups	1	2	1	NA^a^	4
Documents	6	48	24	4	82
Questionnaires	9	3	3	NA^a^	15

^a^*NA* Not applicable

More participant observation was conducted in case 2, given a greater number of team meetings in this setting. In the third case, participants preferred individual interviews rather than a second focus group.

### C & M(s) = > O(s) configurations

Two C & M(s) = > O(s) configurations are proposed by this study. On a first (ontological) level, the context and three mechanisms are identical. On a second (deeper) level, it is the combination of these dimensions that result in two distinct C & M(s) = > O(s) configurations that highlight different outcomes in answer to our research question. Table 3 summarizes the first ontological level components.

**Table 3:** Components of C & M(s) = > O(s) configuration

Configuration	Key components
Context	Decision-making structure
Mechanism	Agency in controversy
Mechanism	Mediation by feedback loops
Mechanism	Mediation by adding intermediaries

The decision-making structure is identified here as context. In fine, context is composed of a structure of interrelated actors that decide on changes to attempt to improve team performance in clinical practice. Otherwise, three mechanisms are conceptualized. Agency in controversy refers to different types of action taken by actors, particularly those in the decision-making structure, when they are involved in a controversy. Mediation, either by feedback loops or by adding intermediaries, is described below. Mediation initiates or arises out of controversy; and tends to transform or maintain structures and their interrelations and to transform professional clinical practice. The following sections present the second level with two type of configurations C & M(s) = > O(s).

### Type 1 C & M(s) = > O(s)

**Fig. 3:**
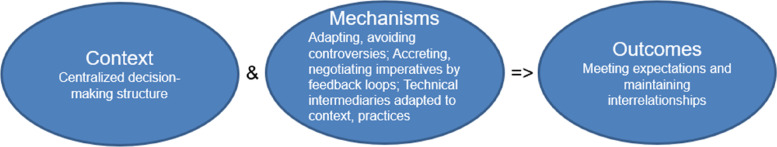
Type 1 C & M(s) = > O configuration

Type 1 C & M(s) = > O(s) configuration presents a context where a decision-making structure is hierarcally centralized. For mechanisms, actors adapt and avoid controversies, especially those in the decision-making structure; they accrete and negotiate imperatives through feedback loops; and, they add technical intermediaries that are adapted to the context and practices. The generated outcomes correspond to expectations and maintain interrelationships. Figure 3 models this configuration.

### Context: centralized decision-making structure

Context, in the Type 1 C & M(s) = > O, is characterized by a centralized decision-making structure. A small group of managers decides on changes that ought to be introduced into clinical practice. In LPGs, local managers present their decisions to clinical team members. One HN, part of the decision-making structure, shared her experience:

You have to decide about an indicator prioritized by the Care Directorate. You have to choose one. We chose it ourselves and then we brought it to the team, saying: ‘We’d like you to choose an indicator with us, but we’ve already kind of chosen it’ (laughter) since we have already prepared an action plan for the pain indicator ... So it was a non-choice ... and we are doing the same thing again with our clinical care standards mandate.

Thus, although the Care Directorate mandated each team to select an indicator from an established list of nursing performance indicators, it appears that this local management team presented an indicator they had already chosen (pain prevention and management), as well as their analysis and identified solutions. In this particular case (case 3), almost 90% of all performance improvement projects (up to 15 concurrent projects) are mandated by the Care or Medical Directorate, who identify crosscutting issues. These managers do not carry out a local evaluation. They propose standardized solutions to local managers who then work together to contextualize the solutions prior to their implementation with unit-based team members. A centralized decision-making structure thus functions at two levels: (a) the institutional managers who decide about 90% of a unit’s projects and (b) the local managers who contextualize these decisions prior to implementation with unit teams.

### Mechanism: Agency in controversies – adapting and avoiding controversies

For the first type 1 mechanism, actors, especially those in the decision-making structure, rarely confront controversies. Controversial projects or some of their components are adapted (e.g., through delay, adjustment, or association with other projects). The purpose of this adaptation is to make clinical sense of these projects, avoid their failure, meet expectations, and prevent burnout.

For example, the LPG needed to identify a priority indicator to work on. An NS explains the choice made by the management team:

We would have liked to work on preparation for discharge. I think that was the one that brought us the most. However, we do not have the conditions to succeed and since we could not choose an indicator that would end in a failure, because we knew that it was already a programmed failure, here we are, we chose another one. In fact, according to the experience of another department for the preparation of discharge, we don't have a secretary who is there all the time, a liaison nurse who is there regularly, the involvement of the doctors throughout the hospital course, and we don’t have this infrastructure, nor this intense collaboration as there can be in medicine with the medical team. Therefore, that is why we knew we were going to fail. In the other department, they couldn’t complete the whole process on discharge preparation.

In fact, the management team did not engage in the controversies related to resources and collaboration with the medical team that would be – in their opinion – factors to adress the priority issue of discharge preparation. The choice of the management team was based on their capability of succeeding, taking into account the actors and their interrelationships (i.e., the context). In other words, they adapted their decisions to the context and avoided controversy. Fourteen months later, the clinical team, and then the head physician, questioned the management team about this issue and a project was implemented.

Another example is that some projects mandated by medical or care management made little sense to the management team. These projects are not discarded; rather, they are adapted. One CNS explains this for the performance indicator selected:

Well then, ‘how are we going to be able to articulate this in the service?’ It is going to require quite a bit of work time on the part of the CNS. We are not going to pay CNSs for something that is not going to bring anything to the service. ‘How are we going to do it?’ In the performance project, we (i.e., the management team) thought about it, and then we finally decided to see if it was possible to combine the two projects so that it would make sense.

In concrete terms, the management team selected all or part of a project and combined it with another project. During the LPG meetings, work on the prevention and management of the pain indicator was linked to five other projects. On the one hand, management team motivated these decisions based on their capability of meeting expectations, of giving meaning to the project, or of acting to improve clinical practice. On the other hand, they repeatedly expressed the view that this strategy avoided the burnout associated with too many projects. The rejection of a project by management was not an option, even if it made little sense for their team or clinical practice. Thus, the management team did not engage in a controversy with management; they adapted the projects to contextualize them, which is a type of agency in controversy.

### Mechanism: mediation through feedback loops – accreting and negotiating imperatives

For the second type 1 mechanism, feedback processes were performed in several loops. These feedback loops allowed, progressively and by accretion, for the negotiation of adaptations (when necessary) and the integration of various propositions for improving clinical practice and to avoid controversy. The priority of the coaching team was to repeat success. A CNS makes these feedback loops explicit to management:

It is always with the NS that we negotiate and who agrees with us on the issues to take to management. It is like the service projects for 2021, we saw them, corrected them, and then we discussed with the NS who then went to the department director of care service with the projects.

The management team conducted several discussions to negotiate solutions. Once the management team was aligned on possible solutions, the NS presented them to the directorates. The directors either did or did not make new proposals that might need to be added by the management team. In the end, directors validated these solutions.

Such loops were observed with the clinical team. The management team presented them with the solutions validated by directors. Progressively, the clinical team made various proposals. The management team accepted some of the proposals and adapted them if necessary. These processes were performed with the repetition of feedback loops on the same theme.

In concrete terms, for the work on the pain indicator, which lasted 13 months, the management team performed more than 10 preparatory or follow-up sessions. Eight LPG meetings were performed with members of the clinical team, not counting the numerous informal exchanges. This work was regularly presented to directors. Proposals for improvement were able to (re)emerge in each meeting and were progressively integrated for the improvement of clinical practice. These feedback loops by accretion allowed mediation of the interrelations between actors and avoidance of controversies: the imperatives of different actors were almost systematically integrated in the projects.

### Mechanism: mediation by adding technical intermediaries adapted to the context and practices

For the third type 1 mechanism, actors developed technical intermediaries to facilitate, support, and ensure the achievement of set objectives. Intermediaries were consistent with or easily integrated into existing clinical practices. Where intermediaries were controversial, they were quickly discarded or improved. Intermediaries included guidelines for computerized patient record documentation, automated pain assessment prescriptions, and pain assessment, prescribing, or management. An HN presents the development of these intermediaries:

The CNS, who built the project and the tools, validated precisely these constructions of data sheets, etc. with our collaborators. Therefore, she came at unplanned times to ask the nurses in the field if it corresponded to their practice.

For the CNS, it was a matter of ensuring that the technical intermediaries developed were consistent with and integrated into current practices. When intermediaries were not controversial, they were integrated into the contexts, interests, and values of the actors. Some technical intermediaries facilitated the rapid achievement of a set objective. We elaborate on this result in the next section.

### Outcomes: meeting expectations and maintaining interrelationships

For the outcomes, one expectation was that 75% of hospitalized patients would receive a pain assessment within 4 h of their admission. Another expectation was that inpatients experiencing pain would receive a pain assessment every 6 h. To meet these expectations, the management team added automated pain assessment prescriptions to computerized patient record. In addition, the management team modified the guideline and informed the clinical team. These initiatives achieved both goals within a month (e.g., the frequency of pain assessment doubled). Their nursing performance indicator scores were in the top 10% of the institution.

For the processes, each LPG meeting was conducted according to the intervention model implemented in the hospital and according to the predefined frequency. Members of the clinical team were present at each meeting. The management team selected a priority indicator and presented an action plan with SMART (specific, measurable, acceptable, realistic, and time-bound) objectives. Processes and results were fully aligned with management expectations.

Some might consider this type 1 configuration to be exemplary. However, almost all professionals on the unit (including managers) questioned whether clinical practice was actually improving. For example, during an observed LPG meeting, one nurse mentioned that pain assessment practices were not similar among nurses on the unit and were sometimes deficient (controversial). The managers proposed to integrate this reflection into a future project (sunset and accretion), as the objectives were met as expected. Three months later, this project had not started. The feedback on clinical team performance system produced two outcomes: (1) maintaining interrelationships among stakeholders and (2) conforming to expectations.

### Type 2 C & M(s) = > O(s)

**Fig. 4:**
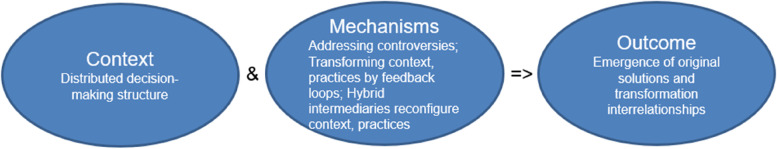
Type 2 C & M(s) = > O configuration

Type 2 C & M(s) = > O(s) configuration presents a context defined by a decision-making structure that is distributed between actors, including clinical teams. Actor performed mechanisms address controversies, transform context practices through feedback loops, and, add hybrid intermediaries that reconfigure context and practices. The generated outcomes correspond to the emergence of original solutions and the transformation of interrelationships. Figure 4 illustrates this configuration.

### Context: distributed decision-making structure

The type 2 context is characterized by a decision-making structure that includes members of management and clinical teams. This structure is extensive and distributed.

Following the departure of several managers and members of the clinical team (40% turnover rate), one team was experiencing a significant dynamic problem. The management team organized numerous meetings with the management and clinical teams in order to resolve it. A NS explains the principles that guided this process:

You start from the principle that the situation is known from the people who are in the base, and it is from the base that you raise the problem. Then you accompany this problem, you elaborate it, you confirm it or not, but really those who have the solution and who know are those who are on the ground. In addition, they are the ones who tell you. Then by telling you, you connect that, so afterwards there are aspects that are theoretical, philosophical aspects.

This NS considers that the clinicians know their clinical reality. He describes a problematization process that is co-constructed between actors, based on their knowledge, and theory to derive a solution. According to him, these principles imply a distributed and extended decision-making structure where priorities, possible solutions ton ongoing problems, and their selection and implementation are discussed and decided collectively. For example, at one meeting, a problem concerning the preparation of medications was highlighted. At the next meeting, a member of the clinical team presented an analysis of the problem and proposed possible solutions. For 20 min, the actors discussed alternative solutions. A project was adapted and it was decided to test it. A clinical team member and an HN produced a summary document. This proposal evolved through ongoing team reflection in action.

### Mechanism: agency in controversies – addressing controversies

For the first type 2 mechanism, certain situations or solutions paths generated controversies that were prioritized and addressed systematically. These controversies involved different actors, roles, practices, interests, and values. They gave rise to emotionally charged exchanges.

For example, a nurse proposed that the HN alone participate in clinical meetings aimed at clarifying the therapeutic project of the persons being cared for, whereas another nurse wanted to participate. The first nurse saw this as a significant time-saver and the second nurse as the realization of a practice related to her role and professional values. The second example is that, with their agreement, patients were placed in the corridor at 9:00 a.m. in order to wait for their departure at 1:00 p.m. This allowed the staff to clean the room and to take care of the patients or to clean the room for a future person. Some professionals found this solution acceptable because the person being cared for gave their consent and it allowed them to save time between a departure and an entry, while others did not consider it a caring and humanistic practice. The third example is that the management team prioritized the work on the team dynamics issue.

This prioritization may have generated controversy with management, who reiterated their expectations for other projects. One NS may have been pressured to prioritize institutional projects:

I go back to my vice-director in this case and bring the arguments to them, and they understand. Whereas 3 months ago, it was, ‘No, now you go ahead and do it’ (laughs), ‘even if you impose it,’ she says, ‘You’re forcing it...Stop listening to them, you’re forcing it!’ and then I say, ‘But I can’t stop listening to them.’ I cannot force it. I can’t impose because...uh...that would be being a little boss.

In this example, the controversy involves the conception of the role of the NS who should or should not impose changes on the clinical team. Imposing or coercing professionals is not consistent with these values. In sum, actors rehearsed exchanges about controversies that involved differing understandings of roles, practices, interests, and values.

### Mechanism: mediation through feedback loops – transforming context and practices

For the second type 2 mechanism, and as we have already illustrated, the feedback processes were performed in several loops. These feedback loops made it possible, progressively and collectively, for the actors to exchange about the controversies and to identify problems and priority causes, solution paths, practices, values, interests, and different roles. They were able to continue individually in an internal reflexive dialogue and generate transformations in the interrelationships.

These collective feedback loops were made possible by several meetings (exceptional or not): (a) weekly meetings (lasting 1 h) between the members of the management team, (b) *a day in the green* for each professional group, (c) *a day in the green* and monthly meetings (lasting 1 h) with the management and clinical teams, and (d) monthly meetings (lasting 30 min to 1 h) with the NS and his director, in addition to numerous individual meetings. The meetings of each professional group allowed for the identification of expectations and solutions, and for emotions to be expressed freely, without offending their colleagues. In a second phase, these teams met collectively, and the emotions and discourse seemed more constructed. One nurse recalls the effects of these meetings:

I think that the meeting we had during the day where we talked about nursing values, etc. I really noticed a significant change in certain colleagues who, for me, were causing problems at the time (smile). However, it is not a problem that was directly solved by actions taken by the management team; that was personal work.

This nurse highlights the fact that a meeting made it possible to initiate the transformation of interrelationships between the actors. Exchanges on values, practices, and the pursuit of personal reflection would have contributed to this transformation. Moreover, this nurse, members of management, and clinical teams considered the feedback spaces to be insufficient. They decided to set up a practice analysis process. These teams met every 2 weeks for 45 min during working hours. This reflexive interpersonal device encouraged participation and mediated the interrelationships of actors; listening and taking into account controversies, roles, practices, interests, and varied values; and the search for collective solutions to improve clinical practice.

### Mechanism: mediation by adding hybrid intermediaries that reconfigure the context and practices

For the third type 2 mechanism, hybrid intermediaries (which combine different types of intermediaries; e.g., values, human expertise, norms, or literary materials) gradually and collectively (re)questioned and then (re)configured collective values, roles, practice, identity, and responsibilities. For example, the objectives of the *green days* were spelled out in a PowerPoint presentation:

(a) Create a shared vision of professional roles in the care team; (b) share and make explicit the individual and collective values that guide their practice; (c) identify gaps between current roles and the shared vision; (d) identify barriers to this gap; and (e) build a foundation for reflection on courses of action.

Hybrid intermediaries supported the achievement of each objective, for example, reading and exchanging specifications, brainstorming on issues or solution paths, and using an Ishikawa diagram to look for root causes, value tables, issues, and prioritized solution paths. These intermediaries aimed to address the issue of team dynamics by linking actors and their roles, practices, interests, and values.

To resolve the controversies, and with the help of these intermediaries, members of the management team questioned the players: “How is our clinical practice? Can it be standardized? What value(s) guide this practice? What is our collective identity? What do we want to transmit to patients or new collaborators about our unit, our practices, and our values? On this chart we have written benevolence – is this practice benevolent?” In addition, they communicated their own values in an engaged dialogue. They actively participated in the negotiation of collective decisions. These questions and intermediaries mediated controversies and allowed for the development of a collective identity.

Subsequently, members of the management team extended the questioning: “When we implement this decision by consensus, how will we respect it and make it respected collectively?” This question was aimed at recognizing the responsibility of each person in the consensus decision. Even more, NS questions tended to develop collective responsibility in their implementation: “We say what we do, we do what we say”. For these members of the management team, each employee is responsible for ensuring compliance with the decisions made by the collective for themselves and for others. In other words, these actors seem to be co-constructing a collective normativity. The actors, their roles, their practices, and their values were interrelated to develop a coherent collective identity and normativity. The practice analysis process allowed for exchanges on the lack of coherence between what was said and what was done. These exchanges led to questions and proposals for follow-up, for example: “How should I approach this problem with my colleague? How many times should I tell him? Whom should I turn to next?” In sum, these hybrid intermediaries mediated actors, identity, and collective responsibility in development.

### Outcomes: emergence of original solutions and transformation of interrelationships

Prioritized by the clinical and management teams, four projects for reorganizing clinical practice were implemented during data collection: transmission at the beginning of the schedule, communication during the schedule, medication preparation, and practice analysis. According to various professionals, two were successfully implemented and two others required adaptations that were under way at the end of the data collection. According to these professionals, these original solutions were aimed at improving team dynamics. In addition, feedback made it possible to address controversies, create feedback loops, and introduce hybrid intermediaries that transformed the interrelationships between certain actors. It has developed the co-construction of a collective identity and normativity.

Feedback on clinical team performance did not follow the model advocated by the institution. A controversy emerged: “Is feedback really being given?” From the director’s perspective, feedback did not meet expectations. The management team did not select a priority indicator, conduct LPGs, or develop a specific action plan. From the management team’s perspective, however, feedback was being given. The team developed a team dynamics questionnaire that provided initial indications. The results of this questionnaire indicated suboptimal performance. Presented to the management and clinical teams, these results led to the work presented here. In short, the emergence of these original processes was, for the clinical and management teams, feedback. The NS makes these differing understandings of feedback explicit:

There’s a way to identify whether or not you have a problem with indicators today. If they tell me pain in the unit is well managed, it is not well managed. What will give the information to those who look at us from the outside are the indicators. If the indicators give good information, I will not necessarily need to work in that area because the indicator is good. However, just because the indicator is good does not mean that my care is good. In my approach, I will have to re-problematize. Because even if the indicators are bad, it is not because the indicators are bad that the care is bad. So [...] I cannot avoid questioning that; but questioning this indicator, in a much broader sense, than just the indicators that are given; I have to broaden it because the phenomenon is much more complex than the one it is reduced to.

Although their nursing performance indicator scores were also in the top 10% of the institution and met expectations, the NS felt that pain was qualitatively not being managed well. He (re)questioned this clinical practice, the feedback model that reduces the complexity of the real world and produces partial information that might be sufficient for some. He proposed a contextual (i.e., relational) problematization related to clinical practice, its values, identity, and normativity.

### Synthesis of C & M(s) = > O(s) configurations of types 1 and 2

In the same institution, based on a single implementation model of feedback, different contexts, practices, and conceptions coexist. They constitute different feedback systems that generate specific outcomes. When JR discussed with certain actors the hypotheses of configurations, different conceptions could be (re)affirmed. For example, when he evoked the strategy developed by one NS (type 2 configuration) to a second NS (type 1 configuration), the second one replied:

“I would not be comfortable if I did not have the support of my hierarchy. Because I could always be seen as the ugly duckling and even if, I find it useful, necessary for my team. I need to have an outside look that validates. ‘Ok I’ll give you a year.’ We negotiate. ‘I don’t agree with what you do, but I let you go and then you give me feedback.’ […] Finally, I also work in Switzerland because everything is negotiated, everything is compromised, and that suits my character better. […] So afterwards, you can be seen as the black sheep, because you do not follow the management’s plans, well that’s possible. However, I would have a little more trouble if we were not supported in this.”

This NS explains how her “Swiss” culture of negotiation and compromise, her identity, and her character configure her practice, the feedback system, and the type 1 C & M(s) = > O(s) configuration.

A synthesis of the type 1 and type 2 C & M(s) = > O(s) configurations is presented in Table 4.

**Table 4:** Synthesis of C & M(s) = > O(s) configurations

Key components	Type 1 configuration	Type 2 configuration
Context Decision structure	Centralized	Distributed
Mechanism Agency in controversies	Adapting and avoiding controversies	Addressing controversies
Mechanism Mediation by feedback loops	Accreting and negotiating imperatives	Transforming context and practices
Mechanism Mediation by adding intermediaries	Technical ones adapted to the context and practices	Hybrid ones that reconfigure the context and practices
Outcomes	Meeting expectations and maintaining interrelationships	Emergence of original solutions and transformation interrelationships

Each type of configuration is presented according to its context, its three concomitant mechanisms, and its specific results, according to the two ontological levels. Each feedback system works. However, it does so differently according to its actors, their interrelationships, and its specific mechanisms. For example, some actors (e.g., directorate, physician managers) are not present in formal feedback meetings. However, they do interact and influence the feedback on clinical team performance system. Their quantitative outcomes (i.e., indicators of nursing performance) seem relatively similar and their qualitative outcomes are different. They are so in the real and the actual, that is, according to the context, the mechanisms, and what they generate as results (real), as well as according to the actors’ conceptions of them (actual). Certain central issues seem to configure them: identity, normativity, and collective practices.

## Discussion

This research produced two C & M(s) = > O(s) configurations that explain how feedback on clinical team performance works, for whom, in what contexts, and for what changes. It highlights different decision-making structures within the same institution – how they configure feedback and how they relate to controversies – and presents controversies that emerge and develop in feedback. It also illustrates how actors and intermediaries mediate, in concomitant actions, the interrelationships in feedback to generate varied feedback outcomes. The outcomes depend on the types of configurations and concern the context and various changes in practices (e.g., automated prescriptions, communication, clinical team dynamics, or medication preparation). The first ontological level – defining context, mechanisms, and outcomes – provides a first explanatory stratum common to the different cases. The second ontological level allows for their specification. In this sense, these strata offer a generalized and transferable explanation (first stratification), as well as a contextualized and specific explanation (second stratification).

This feedback on clinical team performance theory offers original markers for thinking or acting intentionally in feedback systems that are dynamic and polymorphic in nature. For clinical teams and based on the results, we suggest analytical questioning related to the context, mechanisms, and outcomes identified in this research in Table 5.

**Table 5:** Considerations and examples of analytical questioning for C & M(s) = > O(s) configurations

Configurations	Considerations	Examples of analytical questioning
Context	Context configures: • interrelations of actors and intermediaries, • type of action taken or not taken by actors in practice change projects. Clarification of context makes it possible to specify roles and responsibilities of various actors in feedback and clinical practice, their values, and their identity.	• Does the decision-making structure allow for the participation of professionals, to what degree, and for what theme? • What are the associated imperatives or interests (e.g., for interrelationships, feedback, and clinical practice)?
Mechanisms	Controversies are inherently in feedback systems. They involve a variety of values, emotions, interests, actors, practices, identities, and responsibilities.	• How do actors intentionally act in these controversies? • What are the different strategies for facing them or not? • What are the central issues of these controversies and mediations? • How can they be activated in order to reach convergences?
Outcomes	Feedback systems generate outcomes: • some are measured by indicators and others do not, • some generate certain and others uncertain outcomes, • they transform or maintain the interrelations between actors, • they generate paradoxical outcomes: e.g. result of an indicator may be satisfactory, but not the practice, or vice versa.	• What do these intermediaries say about the nature of a problem? • Are they an entry point to a controversy or the definition of the problem? • What is an acceptable or relevant indicator? • To what extent is it shared or negotiated and with whom? • What weight is given to qualitative or quantitative indicators?

In response to the limitations of our rapid realist review, data collection over a 6 month period (in cases 2 and 3) allowed us to better grasp the chronology of events and to explore historical dimensions. It allowed for the articulation of temporal understandings and configurations with reflexive support from various stakeholders (e.g., participants, expert panel, and management team).

More specifically, the configurations of our rapid realist review seem to portray an idealized worldview, independent of the interrelationships of the context or the mechanisms of the real world. In some respects, this has (re)produced an arbitrary simplification of the complex social world [40]. The configurations seem a posteriori to be shaped by the included documents and the researcher’s initial standpoint. In the first example, the rapid realist review assumed a favorable context. Today, we (re)question this conception of favorable context. It seems to have little connection with the real world and the three cases included in this research. Second, the mechanisms identified in the rapid realist review, for example, adjustment of identity, role, values, or clinical practice, are close to those identified in this research. Rearticulated here are the relationships between identity, normativity, and collective practice, central concepts that are mobilized in the mediations (mechanisms). Third, although in the rapid realist review the intermediaries seem to be instrumental or utilitarian tools, in reality, they are mechanisms specifically intertwined with their context and their outcomes. Fourth, the conception of sustainable alignment evoked in our review seems overrated or idealized in a real world in constant transformation. In sum, it is not clear that the two configurations produced in this research articulate those of the rapid realist review. If an articulation were to be identified, it would instead be related to the ongoing problematization of our research object and our efforts to gain depth, realism, and explanatory justice.

### Comparison with existing literature

Brown et al. [41] have identified 30 feedback theories, mainly from the cognitive, behavioral change, psychological, and educational sciences. To our knowledge, there is no theory comparable to the one we are presenting. For example, for some theories such as the social hypotheses refer to the existence of benchmarking, strong communication channels, exchanges, and support by competent people or prioritized or shared objectives [5, 41]. Another example is that political, social, or economic controversies are rarely mentioned. Greenhalgh et al. [9] report that when professionals perceive certain approaches to be political or economic, they are criticized. For the three cases included, current theories do not appear to explain the observed or reported outcomes. Our theory may echo some general explanations or recommendations. However, it also offers new insights through an in-depth explanation of contextualized mechanisms and outcomes produced.

### Strengths and limitations

The results present a theory of feedback that makes explicit the social dimensions and socio-technical and contextual interrelationships. Thus, this theory offers a response to authors who recommend prioritizing a contextualized explanation of interrelationships and underlying feedback mechanisms [5, 6, 9]. Moreover, it identifies central issues of identity, normativity, and collective practice.

The theoretical apparatus and method helped to support our reflections on a complex real world. The results are consistent in the spaces of mechanisms we wished to explore: controversies and mediation. In terms of method, the multiple qualitative case studies and the inclusion of heterogeneous cases offered contrasting and in-depth access to the real world. The five methods of data collection provided dense and complementary material. The repetition of interviews and focus groups, modeling, and discussions with the management team and the panel of experts; the return to theories and transcripts; and the continuous writing of analytical notes in the logbook favored the abduction and then the retroduction of mechanisms. At first glance, the retroduction might seem more complex, given the many intermediaries and actors. However, these interrelationships have contributed to and facilitated the construction of this theory of feedback, including polymorphous practices and conceptions.

Regarding the limitations, other experiences could enhance these results. There are a variety of practices, identities, and collective normativities that configure a feedback system. More time spent on case 1 would have allowed densifying the data on this case and possibly brought new elements or not. Moreover, we did not observe the care provided to patients. Further research could link the configurational typologies to changes in patient care processes or outcomes.

## Conclusions

This critical realist qualitative multiple case study produced a theory, as well as a methodological apparatus, to embrace the complexity of feedback to clinical teams. This research has at least three implications. First, it should not be assumed that there is a shortlist of explanations that can be applied to all contexts. We recommend creating spaces to think and act in contextual transformation of feedback on clinical teams. These spaces should bring together stakeholders. Second, it is essential to develop theories that support reflexive thinking to explain realistically, justly, and critically a complex real world. Third, a democratic debate must address the first two points. It must bring into play the controversies and the possible solutions.

## Supplementary Information

**Additional file 1.**

**Additional file 2.**

**Additional file 3.**

**Additional file 4.**

**Additional file 5.**

## Data Availability

The datasets used and/or analyzed during the current study are available from the corresponding author on reasonable request.

## Abbreviations

ANT: Actor Network-Theory
CHUV: Centre Hospitalier Universitaire Vaudois
C & M(s) = > O(s): Context & Mechanism(s) = > Outcome(s)
CNS: clinical nurse specialist
CR: Critical Realism
HN: head nurse
LPG: local performance group
NS: nurse supervisor
