# Supplementary material for: Feedback on clinical team performance: how does it work, in what contexts, for whom, and for what changes? A critical realist qualitative multiple case study
Source: BMC Health Serv Res. 2023 Apr 27;23:410. doi: 10.1186/s12913-023-09402-x (PMC10136404; doi:10.1186/s12913-023-09402-x)
Supplement: Supplementary file 2 — Additional file 2. [file 12913_2023_9402_MOESM2_ESM.docx]

#
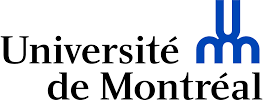
Questionnaire related to professional activity

**Search**

Improving nursing performance: theorizing translation operations in interprofessional team feedback systems

# Researcher

Joachim Rapin, CHUV, PhD student, Faculty of Nursing, University of Montreal, Phone: (*personal number*), Email: [joachim.rapin@umontreal.ca](mailto:joachim.rapin@umontreal.ca)

**You are free to complete or not this questionnaire.**

The student researcher is at your disposal should you need it.

Last Name: First Name:

Institution: Department:

Department/Unit:

Title of your current job at CHUV: Number of Years working in this job:

Highest degree earned (one response only):

- Compulsory schooling
- Learning
- Graduate School (ES)
- Other:
- University of Applied Sciences (UAS)
- Mastery
- Doctorate

Number of months working with the CHUV nursing performance improvement system:

What activities related to the CHUV nursing performance improvement system have you been involved in?

The questionnaire is to be given to the researcher
