# Supplementary material for: Feedback on clinical team performance: how does it work, in what contexts, for whom, and for what changes? A critical realist qualitative multiple case study
Source: BMC Health Serv Res. 2023 Apr 27;23:410. doi: 10.1186/s12913-023-09402-x (PMC10136404; doi:10.1186/s12913-023-09402-x)
Supplement: Supplementary file 3 — Additional file 3. [file 12913_2023_9402_MOESM3_ESM.docx]

## Interview Guide

## Introduction

## Informal welcome upon arrival of participants

## Installation and start-up of the recorder

## Formal welcome

## Personal introduction

## Acknowledgement of participation

## Round table introduction of each participant

## Reminder of the purpose of the research

## Ensure that all questions have been answered

## Make sure you have received all signed information and consent forms, for the 2nd interview make sure you have continuous consent

## Brief introduction (purpose, process and duration)

## How it works

## Reminder of the conditions of participation expected from everyone: commitment to the two sessions, openness during the meetings (to themselves and to each other), values and trust, confidentiality about the participants and the content of the interviews.

## *Only for group interviews*

## *The objective is not to reach a consensus among participants, but to reach a contrasting understanding of the feedback.*

## *Discussion of the research topic among participants, not with the researcher*

## Role of the researcher understand the experience of the participants, ensure that everyone can express themselves freely, at their own pace, and have sufficient time to speak.

## Do you have any questions? Is this information clear enough?

**General question**

Reminder of the research questions, their importance and the research phases

1. How does a NPIS influence feedback system entities and their interactions, in relation to context and over time?
2. How does translation occur in feedback systems, in relation to context and time?
3. How do feedback system entities and their interactions transform a NPIS, in relation to context and over time?

**Specific questions part 1**

*Theoretical parameters and heuristics: System matrix, mechanism to be investigated and others*

| NPIS | Have decisions been made beforehand on NPIS, could you describe their influences on the feedback? How did it evolve? Who was involved (groups and individuals)? Were specific tools used? In what context?  If necessary, the institutional context could be questioned |
| --- | --- |
| Feedback delivery | How does the process of delivering results work?  Who is involved (groups and individuals)? Are specific tools used? In what context? How did it evolve?  If necessary, the process can be compared with the one proposed for implementation |
| Analysis | How is the process of analyzing the results conducted?  Who is involved (groups and individuals)? Are specific tools used? In what context? How did it evolve?  If necessary, the process can be compared with the one proposed for implementation |
| Action plan | How does the process of developing an action plan work?  Who is involved (groups and individuals)? Are specific tools used? In what context? How did it evolve?  If necessary, the process can be compared with the one proposed for implementation |
| Controversies and convergence | During implementation, what challenges did you encounter? How did they evolve? |
| New roles | Have you implemented any new or specific roles in feedback? |
| Strategy implemented to connect entities | How have the interactions, interests of the feedback participants and the interprofessional team evolved? Did you take any specific actions? |
| Distributed actions | Clarify, if necessary, the results of the processes described above. |

Are there other processes that we haven't covered?

**Specific questions part 2 based on (The RAMESES II Project, 2017a)**

*Theoretical parameters and heuristics: C & M(s) => O*

| Outcomes | What were the results of the feedback for you, your team? Example? Are these results the same for everyone (e.g., subgroup)? How do they differ? Why do they differ? If you were to change something about the current processes, what would you change? |
| --- | --- |
| Mechanisms | What do you think generates these results? More specifically, what interactions? |
| Context | What process might explain the results in your unit? Feedback works in a variety of ways in different contexts, what explains the results you describe in this unit? |

If you had to change something about the current processes, what would you change? What else should we know that we haven't mentioned?

**Closing**

## Summarize the key elements

## Do you have any comments or thoughts that have not been addressed?

## Reminder of the researcher's availability if additional information is needed

## Check on the emotional state of the participants

## Thank you

## Timetable and information on how the next meeting will take place

## The recorder is turned off when the last participant leaves
