# Supplementary material for: Feedback on clinical team performance: how does it work, in what contexts, for whom, and for what changes? A critical realist qualitative multiple case study
Source: BMC Health Serv Res. 2023 Apr 27;23:410. doi: 10.1186/s12913-023-09402-x (PMC10136404; doi:10.1186/s12913-023-09402-x)
Supplement: Supplementary file 4 — Additional file 4. [file 12913_2023_9402_MOESM4_ESM.docx]

## List of codes and their definitions

| **Code** | **Definition** |
| --- | --- |
| Location | Description of the place where an observation takes place |
| Use location | Description of a process related to a place |
| Registration | Intermediate which is a literary inscription of the type: documents, report, guide, dashboard, analysis support, written action plan |
| Artifacts | Intermediary which is a sociotechnical artifact of the type: machine, computer program, software, interactive computer reports |
| Human | Intermediary who is a human being, and also: their skills, knowledge or know-how |
| Resource | Intermediary that is a resource of the type: material or financial |
| Hybrid Intermediate | An intermediary that would combine one or more of the following: literary inscription, sociotechnical artifact, human being or resource |
| Actor | Entity that associates intermediaries, for example: an organization, a human or non human association |
| Mediator | A mediator is able to move other entities. It changes and creates connections that transform the networks |
| Network | A network is composed of actors or intermediaries |
| Controversy | Competing representations of a situation within a given context |
| Convergence | Connection of entities to its problematization and expansion of the network |
| Strategy | Strategy to connect entities to one's problematization in order to expand one's network, e.g. negotiation, creation of new role. |
| Distributed stock | Actions revealed by an individual or a group, however, they are distributed by the interactions within the feedback system |
| New role | Appearance of a new actor, role or interest |
| Transformation | Transformation of connections, processes, entities, feedback system or NPIS |
| Emergence | Appearance of a new process, event, connection |
| Chronology | Element related to time, to chronological evolution |
| 1. Hypothesis | NPIS partially determines the extent to which feedback can engage interprofessional teams to improve their nursing performance. |
| 1. Hypothesis | Feedback to interprofessional teams potentially generates, through problematization, convergence or alignment, mobilization of entities into distributed action to improve their nursing performance |
| 1. Hypothesis | The actions of the actors in the feedback system partially determine the extent to which the NPIS transforms. |
| - 1. Semi-reg. | Appropriate choice of indicators and targets |
| - 1. Semi-reg. | Appropriate choice of a method for transmitting indicator results |
| - 1. Semi-reg. | Understandable information |
| 1.4 Semi-reg. | Pre-existence of dense connections within NPIS networks |
| 1.5 Semi-reg. | Other potential semi-regularity related to hypothesis 1 |
| 2.1 Semi-reg. | Recognition of problems |
| 2.2 Semi-reg. | Activation of values |
| 2.3 Semi-reg. | Introduction of additional information |
| - 1. Semi-reg. | In-depth conversations and critical reflection |
| - 1. Semi-reg. | Agreement about the values inherent in the feedback processes |
| - 1. Semi-reg. | Compromise about the (best) plan to improve clinical practice |
| 2.7 Semi-reg. | Sustainable adjustment and alignment of goals and priorities |
| 2.8 Semi-reg. | Other potential semi-regularity related to hypothesis 2 |
| 3.1 Semi-reg. | Feedback contributes to the transformation of the NPIS through an extension of intersystem mobilization |
| 3.2 Semi-reg. | Other semi-regularity related to hypothesis 3 |
