# Supplementary material for: Feedback on clinical team performance: how does it work, in what contexts, for whom, and for what changes? A critical realist qualitative multiple case study
Source: BMC Health Serv Res. 2023 Apr 27;23:410. doi: 10.1186/s12913-023-09402-x (PMC10136404; doi:10.1186/s12913-023-09402-x)
Supplement: Supplementary file 5 — Additional file 5. [file 12913_2023_9402_MOESM5_ESM.docx]

## Observation guide [inspired by Decuypere (2019)]

| **Date and time** |  | **Location** |  |
| --- | --- | --- | --- |
| **System matrix** | | | |
| **Observed process(es)**  **Context** | **Entities** | **Connections** | **Role and interests** |
|  |  |  |  |

| **Space for mechanisms to be investigated** | | | | |
| --- | --- | --- | --- | --- |
| **Controversy** | **Convergence** | **Strategy implemented to connect entities** | **Actions distributed** | **New role** |
|  |  |  |  |  |
| **Other: transformation or emergence** | | | | |
|  | | | | |
| **Questions** | | | | |
|  | | | | |
| **Reflections** | | | | |
|  | | | | |
